# Epitokous metamorphosis, reproductive swimming, and early development of the estuarine polychaete, *Neanthes glandicincta* Southern, 1921 (Annelida, Nereididae) on the east coast of the Malay Peninsula

**DOI:** 10.3897/zookeys.1011.59780

**Published:** 2021-01-18

**Authors:** Siti Syazwani Azmi, Yusof Shuaib Ibrahim, Saowapa Angsupanich, Pornsan Sumpuntarat, Masanori Sato

**Affiliations:** 1 Institute of Oceanography and Environment, Universiti Malaysia Terengganu, 21030, Kuala Nerus, Terengganu, Malaysia; 2 Faculty of Science and Marine Environment, Universiti Malaysia Terengganu, 21030, Kuala Nerus, Terengganu, Malaysia; 3 Marine and Coastal Resources Institute, Prince of Songkla University, Hat Yai, Songkhla 90112, Thailand; 4 Research Field in Science, Science and Engineering Area, Kagoshima University, 1-21-35 Korimoto, Kagoshima 890-0065, Japan

**Keywords:** Kuala Ibai, natatory chaetae, pelagic larvae, Songkhla Lagoon, South China Sea, trochophore

## Abstract

The reproductive and developmental characteristics of the nereidid polychaete, *Neanthes glandicincta* Southern, 1921, commonly recorded in tropical estuaries in the Indo-West Pacific, were examined from Malaysia (the mangrove area of Kuala Ibai, Terengganu) and Thailand (the Lower Songkhla Lagoon) on the east coast of the Malay Peninsula. Epitokous metamorphosis of fully mature males and females and their reproductive swimming behaviour were recorded based on ten Malaysian epitokous specimens, which were collected at night during spring tides in a period of January 2018 to March 2019. Six Thailand epitokes were obtained in February and March 2006 by the laboratory rearing of immature worms. Epitokous metamorphosis is characterised by the enlargement of eyes in both sexes, division of the body into three parts and modification of parapodia with additional lobes in the mid-body of males, and replacement of atokous chaetae in the mid-body by epitokous natatory chaetae, completely in males and incompletely in females. The diameter of coelomic unfertilised eggs in females was 100–140 µm. After fertilisation, each egg formed a jelly layer, inside which embryonic development progressed. Trochophores hatched out of the jelly layer, entering a short free-swimming larval phase followed by demersal life at the early stage of 3-chaetiger nectochaeta one day after fertilisation. Then, the larvae entered benthic life as juveniles, crawling on the bottom, at the late stage of 3-chaetiger nectochaeta two days after fertilisation. The results indicate that *N. glandicincta* has an annual life cycle, which is usually completed within an estuary with limited larval dispersal ability.

## Introduction

The nereidid polychaete, *Neanthes glandicincta* Southern, 1921 (type locality near Calcutta, India) is commonly reported from Asian tropical estuaries in Indo-Western Pacific Oceans (Fauvel 1932, 1939, 1953; Wu 1967; Wu et al. 1985; Lee and Glasby 2015; Ibrahim et al. 2019). Lee and Glasby (2015) synonymised *Ceratonereis burmensis* Monro, 1937 (type locality: Maungmagan, Burma, and off Bombay, India) with *N. glandicincta*, and also described *N. wilsonchani* Lee & Glasby, 2015 from Singapore, which is similar to *N. glandicincta* but distinguishable by the number of paragnaths. Ibrahim et al. (2019) established the *Neanthes glandicincta* species complex, which included these two species. Hsueh (2019) described *N. kaomeiensis* from Taiwan, which is similar to both *N. glandicincta* and *N. wilsonchani* but differs from them in the absence of a notopodial prechaetal lobe in posterior chaetigers, and thus it is regarded as the third species of the *Neanthes glandicincta* species complex. Lee and Glasby (2015), Sato (2017), and Ibrahim et al. (2019) suggested that several more cryptic species belonging to this species complex may be distributed in the South China Sea and East China Sea coasts.

Reproductive and developmental modes of nereidids are conspicuously variable even among morphologically similar congeneric species (Sato 1999, 2017), although all nereidids are semelparous (breeding only once in a lifetime) (Olive 1983). Many nereidids show swarming behaviour (mass-swimming of sexually mature adults) to shed gametes freely into the water, accompanied by a common set of drastic morphological changes (known as epitokous metamorphosis) into epitokes of the ‘heteronereis form’ in both males and females or males only. These changes include marked enlargement of the eyes, division of the body into two or three parts, enlargement and/or modification of the parapodial ligules and cirri with the addition of some lappets (most marked in the middle or posterior body), and replacement of atokous chaetae by paddle-like natatory chaetae (Clark 1961; Schroeder and Hermans 1975). However, some species spawn without any epitokous metamorphosis and swarming (Sato 2017). These previous findings indicate that reproductive and developmental characteristics may be useful to distinguish morphologically similar but distinct species.

Fauvel (1932) found 'subepitokous' males of *N. glandicincta*, which were collected from Vizagapatam, India from May to June 1926; they were on the way to epitokous metamorphosis, with the dorsal cirri crenate, and with atokous chaetae mixed with the paddle-like natatory chaetae. Later, Fauvel (1939) described epitokous males, which had the body divided into three parts, with epitokous modification in the middle part (beginning at chaetiger XX), based on specimens collected in Singapore (plankton sample) probably during their reproductive swimming. Monro (1937) reported typical epitokous metamorphosis in several males of this species based on part of the *C. burmensis* type material collected from off Bombay, describing the eyes as markedly enlarged, the body divided into three parts, the epitokous modification of parapodia occurring in the middle half of the body (beginning at chaetiger XXI), with the anterior and posterior parts remaining unmodified. It should be noted that both Fauvel (1939) and Monro (1937) reported the epitokous metamorphosis of only males, not referring to that of females.

On the other hand, Wu et al. (1985) described the epitokous metamorphosis of this species (as *C. burmensis*) as follows: eyes were enlarged, the body was divided into two parts, and the epitokous modification of parapodia occurred in the posterior body (beginning at chaetiger XIV) in a male (benthic sample), whereas eyes were enlarged, the body was divided into three parts, the inconspicuous epitokous modification of parapodia occurred in the middle body (chaetigers XVI–XXXIII), and a cleft was present on the anterior margin of the prostomium in a female (plankton sample).

Lee and Glasby (2015) described the epitokous morphology of both *N. glandicincta* and *N. wilsonchani* based on the epitokes obtained from the sediment samples collected from the mudflats in Singapore in a period of December to April, even though some of their materials seemed not fully matured. They concluded that there were no significant differences in the epitokous metamorphosis between the two species or between the sexes of each species, judging that the observed differences between them appeared to be related to the degree of maturity of the specimen. There is no previous report on the early development of any species from the *Neanthes glandicincta* species complex.

It is important, therefore, to clarify the epitokous metamorphosis of both sexes of *N. glandicincta* and their reproductive and developmental characteristics based on the fully mature adults, in order to reveal unknown cryptic species that have been confused with *N. glandicincta*.

In the present study, we obtained fully mature swimming adults of *N. glandicincta* from two localities in Malaysia and Thailand along the east coast of the Malay Peninsula during one-year monthly night sampling in the field and from laboratory culture, respectively. Using this material of *N. glandicincta*, we describe the epitokous metamorphosis of fully mature males and females, their reproductive swimming behaviour, and early development.

## Materials and methods

**Figure 1.**
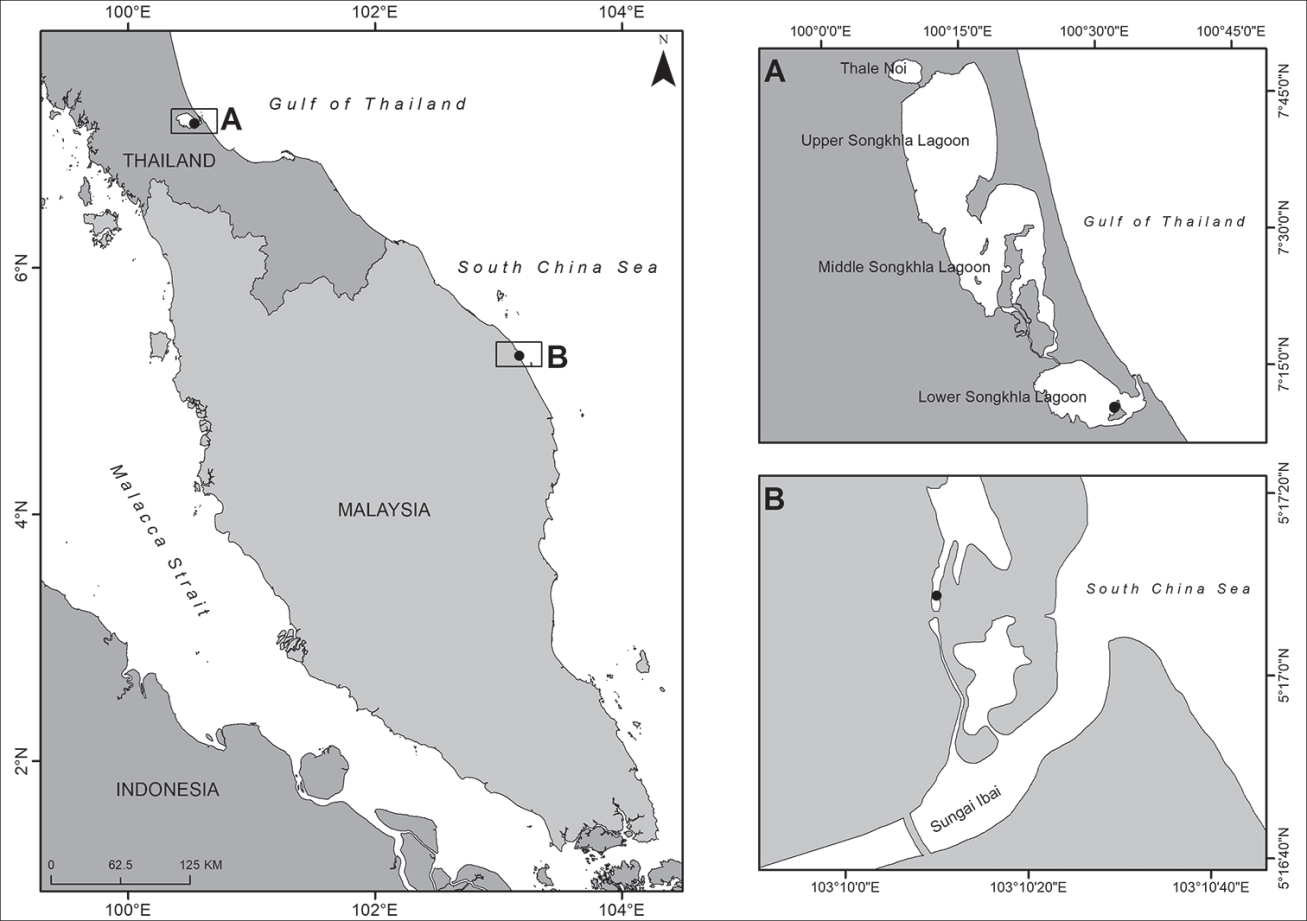
Map showing the collection sites (closed circles) in two estuaries on the east coast of the Malay Peninsula **A** lower Songkhla Lagoon, Thailand **B** mangrove area in Kuala Ibai branched from Sungai Ibai in Terengganu, Malaysia.

At Aowsai in the Lower Songkhla Lagoon (Outer Songkhla Lake) (old name, Thale Sap Songkhla) in Thailand (Fig. 1A), immature worms of *N. glandicincta* were collected from the sediment samples dug out from intertidal or shallow subtidal bottoms using shovels in 2005 and 2008. For atokous morphology observation, some of them were fixed in 10% formalin and later transferred to 80% ethanol for preservation. The other worms were reared in indoor cement ponds (length 270 cm, width 176 cm, height 70 cm) containing coarse sand (10 cm thick) and seawater diluted to a salinity of 15 psu to obtain sexually mature adults. The ponds were maintained with aeration and fed on commercial dry food for shrimps.

Spawning occurred at midnight on 28 February and 2 March 2008 in the ponds. After the spawning of mature adults, all spent worms were fixed in 10% formalin and later transferred to 80% ethanol for preservation. The successfully fertilised eggs, which were obtained independently from three pairs of male and female epitokes in the ponds, were transferred to plastic bottles (diameter 8 cm, height 30 cm; wrapped in black plastic sheets) containing fresh diluted seawater of 15 psu salinity with aeration for embryonic development observation. Swimming trochophore larvae were transferred to glass jars (diameter 22 cm, height 35 cm; wrapped with black plastic sheets at the lower part) containing fresh diluted seawater for larval development observation. A few of developing embryos and larvae were periodically taken out from the plastic bottles and glass jars using a pipette to a glass slide for microscopy. All the development observations were carried out at room temperature (25–30 °C).

Monthly night samplings were carried out in the mangrove area in the estuary of Kuala Ibai, Terengganu, Malaysia, with 365 kilometres distance from the Thailand site (Fig. 1B). The survey was conducted 2–8 h after sunset, around the new or full moon in the period of January 2018 to March 2019. An underwater lamp was used as an artificial light to attract swimming worms. In total, ten epitokes of *N. glandicincta* (9 males, 1 female) were collected with a scoop net as they were swimming in the water surface. In-situ parameters, including salinity and temperature, were measured using Hydrolab Multiparameter. Live specimens were brought to the laboratory for sex determination by examining the coelomic contents (oocytes or sperm) and were fixed in 80% ethanol for preservation.

The maximum body width (BW), excluding the parapodia within chaetigers X–XXX was measured for each specimen. The body length (BL) of the complete specimens was measured from the base of the antenna to the end of the body, excluding anal cirri, and the total number of chaetigers were also counted. The paragnaths on the proboscis were counted in each area. The first and last natatory chaetigers in the middle body of epitokous males were determined by the appearance/disappearance of lamellae at the upper and lower portions on the base of the ventral cirri.

Photographs were taken with digital cameras (Nikon D3400, Nikon FDX-35, Touptek Photonics Toupcam E32SPM) on stereomicroscopes (Olympus SZX7, Olympus SZX16) and compound microscopes (Leica DM300, Nikon Eclipse E600). In some cases, several photographs were stacked to improve the depth of field using a software of Touptek Photonics Toupcam E32SPM. Drawings were prepared with a camera Lucida attached to the microscopes. The ArcGIS 10.3 software was used to prepare the map.

The usage of the nereidid morphology terminology is according to Villalobos-Guerrero and Bakken (2018).

The rainfall and air temperature dataset were obtained from the Malaysian Meteorological Department of the Environment and Water Ministry Malaysia.

Specimens were deposited at the South China Sea Repository and Reference Centre of Universiti Malaysia Terengganu, Malaysia (**UMT**), and the Phuket Marine Biological Centre, Phuket, Thailand (**PMBC**).

## Results

### Taxonomic account

#### Neanthes glandicincta

Taxon classificationAnimaliaPhyllodocidaNereididae

(Southern, 1921)

99854609-E26A-562B-BE87-906BEF3BC279

**Figure 2.**
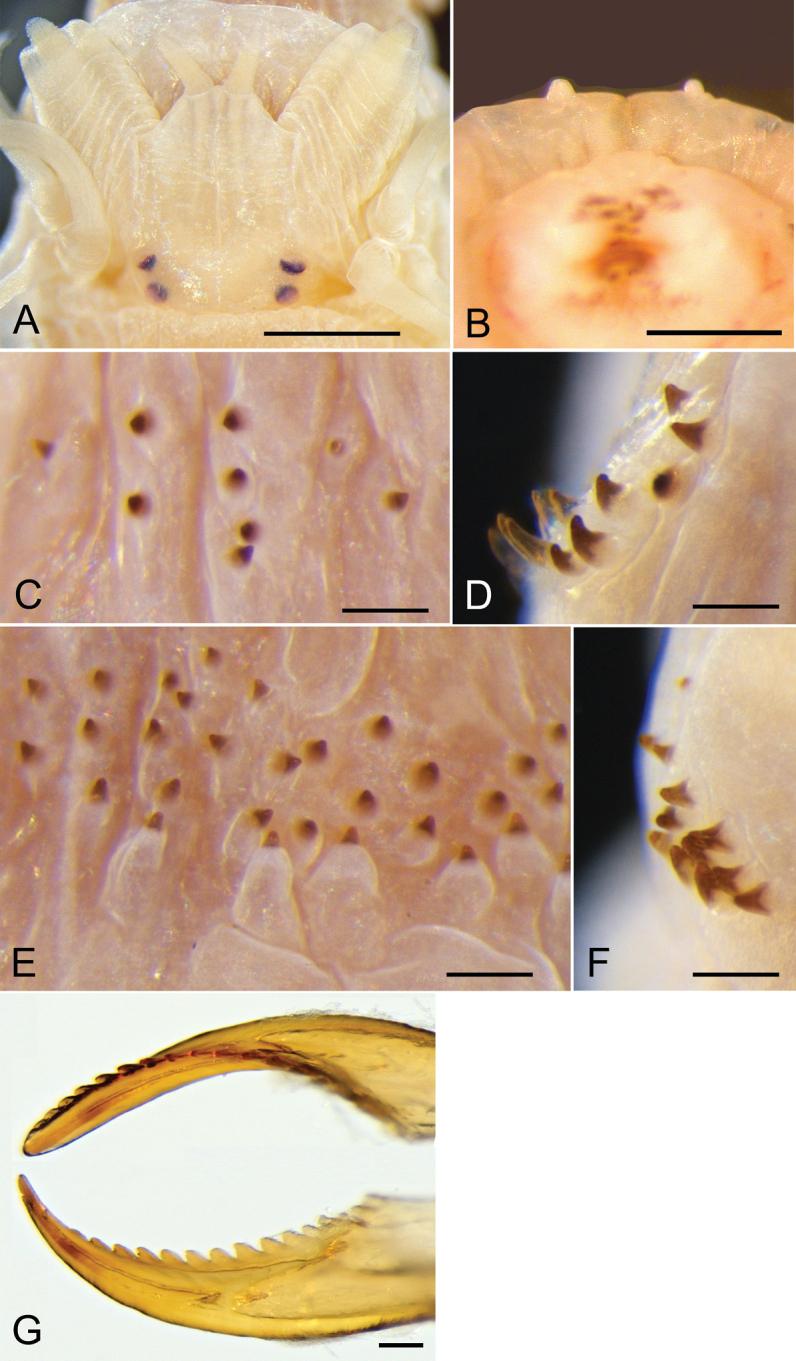
Atokes (**A–F**) and an epitoke (**G**) of *Neanthes glandicincta* (Southern, 1921) collected from the Lower Songkhla Lagoon, Thailand **A** prostomium of an atoke (ind. no. 10 with BW of 1.7 mm, PMBC 21209) **B** anterior view of an everted proboscis, showing a pair of small nipple-like round papillae on area VI in an atoke (ind. no. SL-2 with BW of 1.5 mm, PMBC 21212) **C–F** paragnaths in areas I (**C**), II (anterior and middle parts of left side, **D**), III (central part, **E**), and IV (right side, **F**) of an atoke (ind. no. 1 with BW of 2.3 mm, PMBC 21209) **G** dorsal (upper) and ventral (lower) views of the right jaw of a male epitoke (ind. no. 3M with BW of 1.2 mm, PMBC 20732).

**Figure 3.**
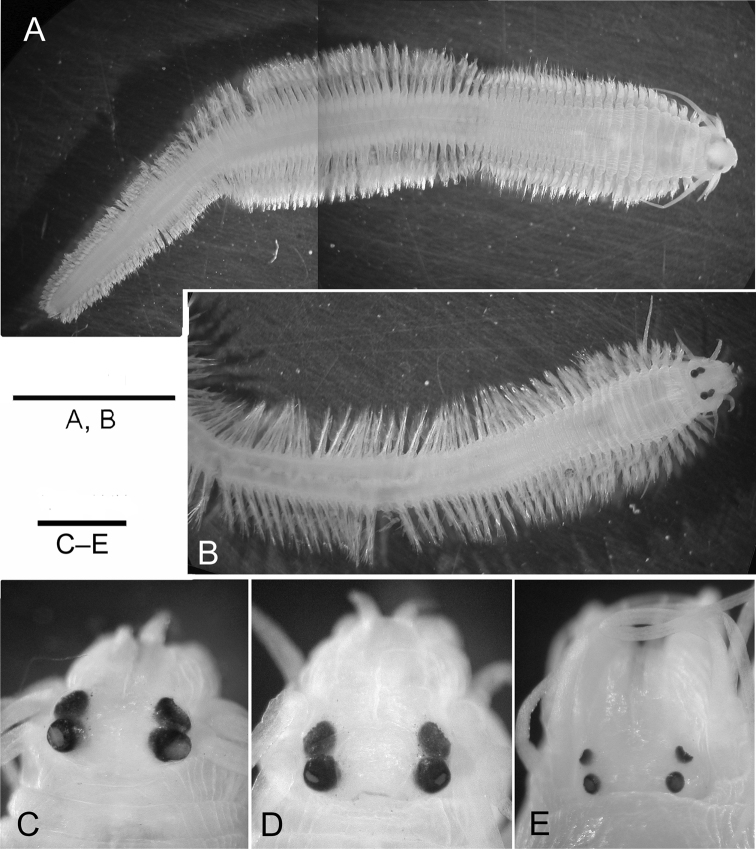
Epitokous males (**A, C**) and females (**B, D**) of *Neanthes glandicincta* (Southern, 1921) collected from the Lower Songkhla Lagoon, Thailand (PMBC 20732) in comparison with an atoke from the same locality (E) (PMBC 21209) **A** dorsal view of the whole body of a male **B** dorsal view of the anterior body of a female **C–E** enlargement of anterior dorsal end of a male epitoke (**C**), a female epitoke (**D**), and an atoke (**E**). Scale bars: 5 mm (**A, B**); 0.5 mm (**C–E**).

**Figure 4.**
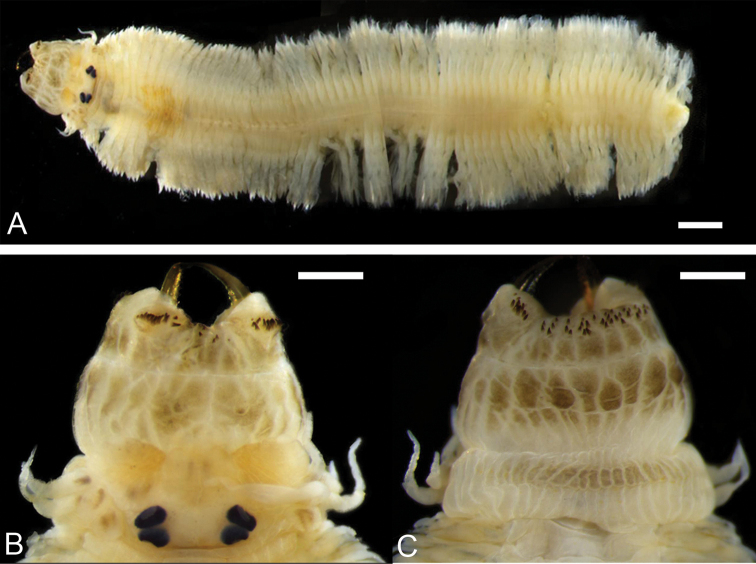
Male epitoke of *Neanthes glandicincta* (Southern, 1921) collected from Kuala Ibai, Malaysia (UMTAnn 453) **A** dorsal view of the whole body (incomplete, with the pre-natatory and natatory regions) **B** dorsal view of the proboscis with pigmentation **C** ventral view of the proboscis with pigmentation. Scale bars: 1 mm (**A**); 0.5 mm (**B, C**).

**Figure 5.**
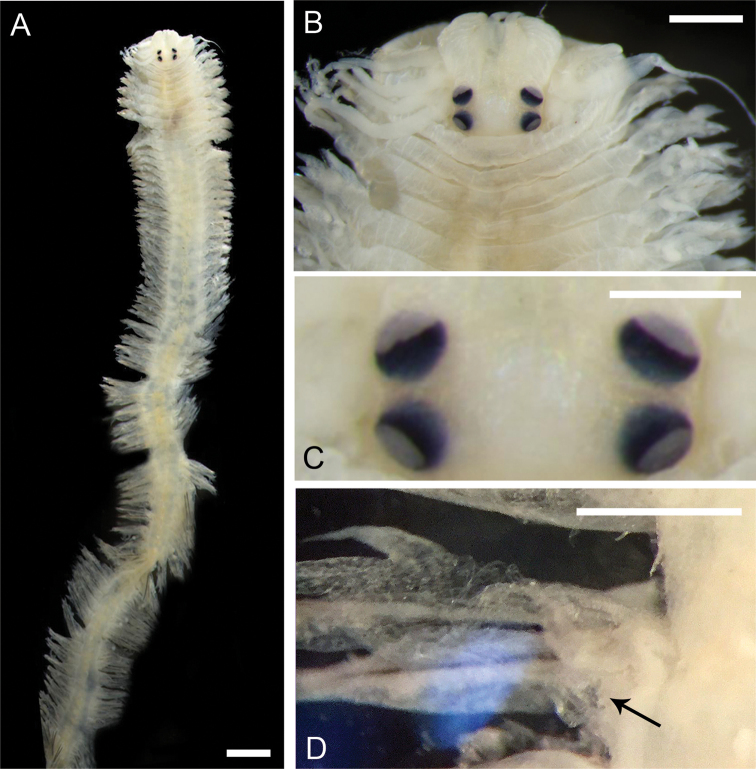
Female epitoke of *Neanthes glandicincta* (Southern, 1921) collected from Kuala Ibai, Malaysia (UMTAnn 449) **A** dorsal view of the whole-body **B** enlargement of anterior end **C** enlargement of eyes **D** rupture of body wall at the ventral surface in the posterior body (arrow). Scale bars: 1 mm (**A)**; 0.5 mm (**B–D**).

**Figure 6.**
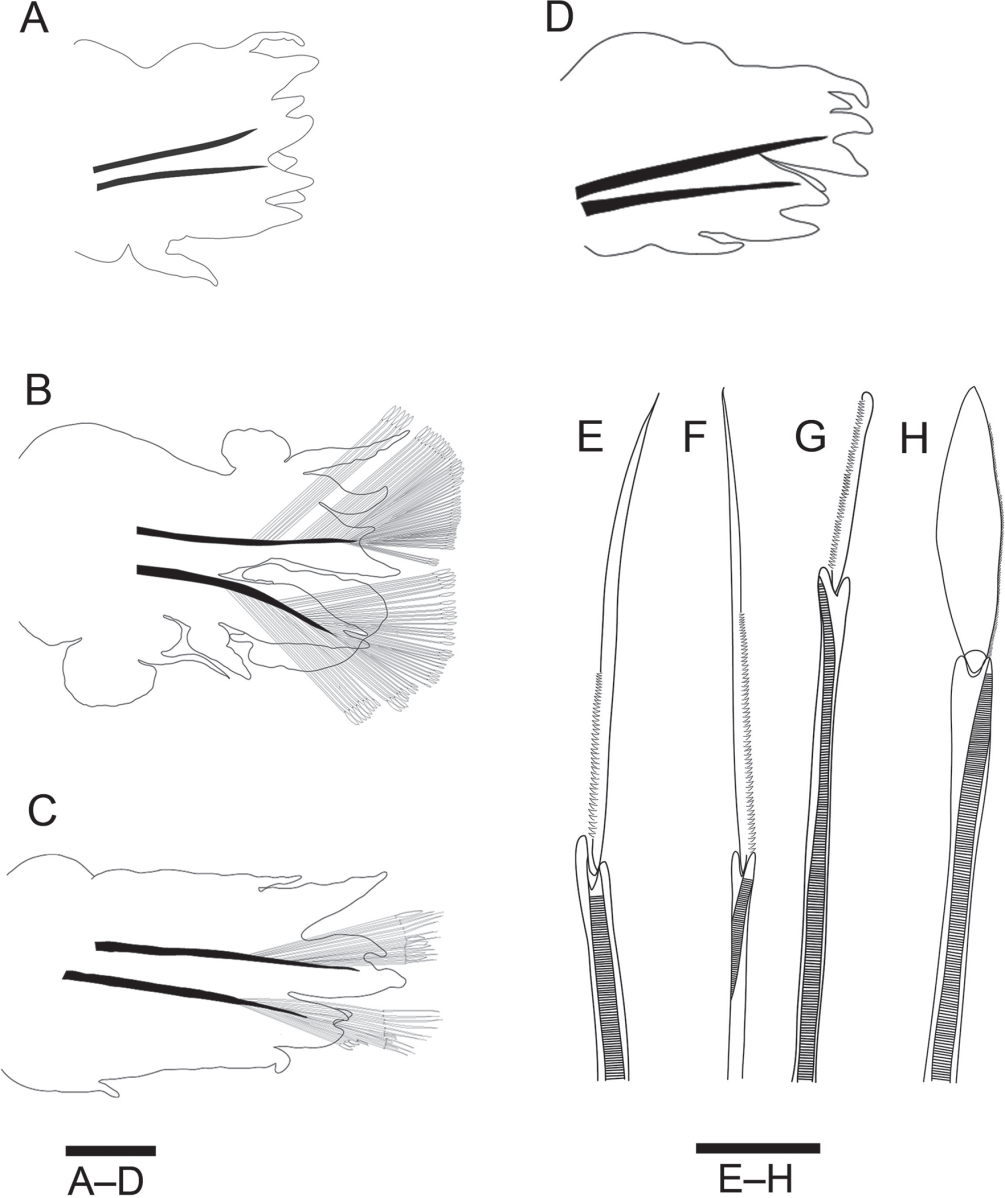
Drawings of epitokes of *Neanthes glandicincta* (Southern, 1921) collected from Kuala Ibai, Malaysia **A** posterior view of the right parapodium 8 in the pre-natatory region of a male (UMTAnn 453) **B** posterior view of the right parapodium 35 in the natatory region of a male (UMTAnn 445) **C** anterior view of the left parapodium 35 of a female (UMTAnn 449) **D** posterior view of the right parapodium 66 in the post-natatory region of a male (UMTAnn 446) **E** heterogomph spiniger from the lower neurochaetae in chaetiger 8 of a male (UMTAnn 453) **F** homogomph spiniger from the upper neurochaetae in chaetiger 8 of a male (UMTAnn 453) **G** heterogomph falciger from the lower neurochaetae in chaetiger 36 of a female (UMTAnn 449) **H** epitokous natatory chaeta from the neuropodium of chaetiger 36 of a male (UMTAnn 453). Scale bars: 1 mm (**A–D**); 0.05 mm (**E–H**).

**Figure 7.**
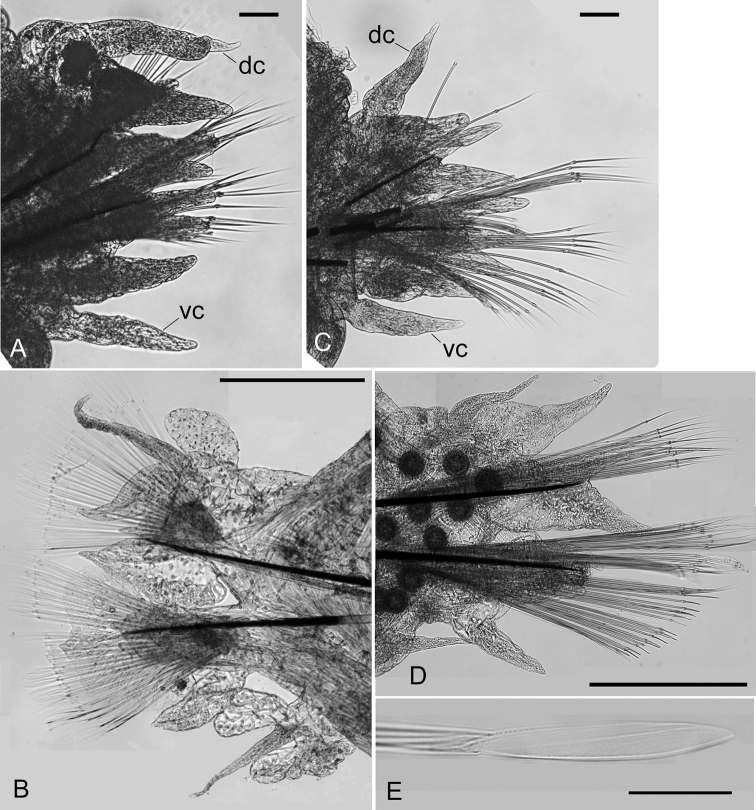
Epitokous males (**A, B, E**) and a female (**C, D**) of *Neanthes glandicincta* (Southern, 1921) collected from the Lower Songkhla Lagoon, Thailand (PMBC 20732) **A** anterior view of left parapodium of chaetiger 5 in the pre-natatory region of a male epitoke **B** anterior view of right modified parapodium of chaetiger 34 in the natatory region of the same male as (**A**) **C** posterior view of right parapodium of chaetiger 3 of a female epitoke **D** posterior view of right parapodium of chaetiger 37 of the same female as (**C**) **E** enlargement of an epitokous paddle chaeta of another male epitoke. Abbreviations: dc, dorsal cirrus; vc, ventral cirrus. Scale bars: 0.1 mm (**A, C**); 0.5 mm (**B, D**); 0.05 mm (**E**).

Figs 2 , 3 , 4 , 5 , 6 , 7

Nereis (Nereis) glandicincta Southern, 1921: 589–593, text fig. 5a–e, pl. 23, fig. 9A–L.
Nereis glandicincta : Fauvel 1932: 92–93; Fauvel 1939: 314–315, Fauvel 1953: 181–182, fig. 91f–h.
Neanthes glandicincta : Lee and Glasby 2015: 80–85, figs 7–9; Misra 1999: 161–162; Ibrahim et al. 2019: 86–89, figs 3, 4.
Ceratonereis burmensis Monro, 1937: 532–536, fig. 1a–f; Misra 1999: 149; Ng et al. 2011: 426.
Nereis (Ceratonereis) burmensis : Fauvel 1953: 196–197, fig. 97d–f.
Ceratonereis (Composetia) burmensis : Hartmann-Schröder 1985: 49 (list); Chan 2009: 165–167, fig. 5a–r, in part.
Not Neanthes glandicincta: Wu et al. 1985: 150–151, fig. 84; Wu et al. 1985: 174–177, figs 98, 99 (described as Ceratonereis burmensis).

##### Type locality.

Brackish lakes or pools at four localities in Barantolla, Dhappa, and Garia near Calcutta in India (Southern 1921).

##### Material examined.

***Sexually fully mature specimens (epitokes)*.** Epitokes collected during reproductive swimming in the mangrove area of Kuala Ibai (5°17'7.6"N, 103°10'10.3"E), Terengganu, Malaysia: 2 males (BW, 1.6–1.9 mm; UMTAnn 445–446), coll. YS Ibrahim, 31 January 2018; 2 males and one female (2.1–2.4 mm; UMTAnn 447–449), coll. YS Ibrahim, 28 February 2018; 3 males (2.2–3.2 mm; UMTAnn 450–452), coll. SS Azmi, 6 January 2019, 2 males (2.1–2.6 mm; UMTAnn 453–454), coll. SS Azmi, 21 January 2019.

Epitokes obtained by rearing immature atokes collected from Aowsai in the lower reaches of Songkhla Lagoon (7°10'37.4"N, 100°32'26.2"E), Thailand (PMBC 20732): 3 males (BW, 1.2–2.1 mm) and 3 females (BW, 1.5–1.7 mm), coll. P Sumpuntarat, 2 March 2006.

***Atokous specimens collected from the same locality as the epitokes*.** Immature atokes collected from Aowsai in the lower reaches of Songkhla Lagoon, Thailand (as above): 5 specimens (BW, 1.6–2.5 mm; PMBC 21209), coll. P. Sumpuntarat, during the period from September to December 2005; 4 specimens (BW, 1.2–1.3 mm; PMBC 21211), coll. S Angsupanich et al., 1 March 2008; 1 specimen (BW, 1.5 mm; PMBC 21212), coll. S Angsupanich, 19 November 2008.

##### Description of atokes.

Ten atokes, including six complete specimens, 27–85 mm BL (Mean ± SD: 60.8 ± 19.7, n = 6), 1. 2–2.5 mm BW (1.7 ± 0.5, n = 10), with 86–122 chaetigers (108.3 ± 15.8, n = 4) (Table 1). Colour in preserved specimens whitish cream (Fig. 2A).

Two pairs of eyes arranged trapezoidally (anterior pair with space wider than that of posterior pair); anterior pair reniform; posterior pair round; two pairs of eyes almost same in size (Figs 2A, 3E). Approximately ten transverse grooves conspicuous in each of massive palpophores. Apodous segment (peristomium) with four pairs of tentacular cirri of unequal length; posterodorsal tentacular cirri longest, reaching back to chaetigers V–VII.

Proboscis with pair of semi-transparent amber jaws, each with ca. ten teeth. Typical conical paragnaths present on maxillary ring (Fig. 2C–F); number of paragnaths and their arrangement on each area of everted proboscis as follows (Table 1): area I: 4–11, scattered and unequal (Fig. 2C); area II: 12–17, in two arched rows, markedly large paragnaths with sharply tapering and curved tip present in anterior and middle positions (Fig. 2D); area III: 38–55, in three or four rows of transversely elongated bands, each paragnath with papilla-like base (Fig. 2E); area IV: 10–14, in triangular patch with markedly large paragnaths present in middle and posterior positions (Fig. 2F). Oral ring with no or few minute paragnaths; number of paragnaths on each area are as follows (Table 1): area V: none; area VI: none or single minute paragnath present, seated on tip of each papilla (usually pair of small nipple-like round papillae visible in right and left of area VI; Fig. 2B); area VII–VIII: none or single minute paragnath present. Total number of paragnaths 94–119.

Uniramous parapodia of first two chaetigers without notoacicula. In following biramous parapodia, notopodia consisting of dorsal cirrus and three ligules/lobe (dorsal ligule, prechaetal lobe and median ligule) throughout. Neuropodia consisting of four ligules/lobes (superior lobe, inferior lobe, postchaetal lobe, ventral ligule) and ventral cirrus in anterior and middle body; superior lobe absent in posterior body (from chaetiger L).

Notochaetae consisting of homogomph spinigers throughout. Upper neurochaetae including homogomph spinigers with long blades and heterogomph spinigers with short blades throughout; some or most of heterogomph spinigers replaced by heterogomph falcigers in middle body. Lower neurochaetae include heterogomph spinigers with long blade (at upper position) and heterogomph spinigers with short blade (at lower position) throughout; some or most of heterogomph spinigers with short blades replaced by heterogomph falcigers in anterior-mid body (from chaetigers XI–XIX usually). Heterogomph falcigers with finely serrated slender blades; few heterogomph falcigers rarely (two of ten specimens) present in lower neurochaetae of chaetiger 1. Conspicuous glandular patches present in dorsal ligules.

Coelom of three individuals filled with many oocytes with maximum diameter of ca. 100 µm.

##### Description of epitokes.

Twelve males, including eight complete specimens, 17–43 mm BL (Mean ± SD: 32.4 ± 8.3, n = 8), 1.2–3.2 mm BW (2.2 ± 0.5, n = 12), with 62–123 chaetigers (100.9 ± 19.4, n = 8). Four females, including three complete specimens, 25–34 mm BL (28.7 ± 4.7, n = 3), 1.5–2. 4 mm BW (1.8 ± 0.4, n = 4), with 84–116 chaetigers (102.0 ± 16.4, n = 3). There was no significant difference in BL, BW, and the number of chaetigers between males and females (Wilcoxon-Mann-Whitney test, P > 0.2). Live spent worms after spawning semi-transparent; live females with greenish eggs.

Two pairs of eyes enlarged in both males (Figs 3C, 4A, B) and females (Figs 3B, D, 5A–C) in comparison with those in atokes (Fig. 3E); enlargement of eyes more remarkable in males than females; two pairs of eyes almost same in size, shape (round or ovoid) and space between right and left eyes. Apodous segment with four pairs of tentacular cirri of unequal length; posterodorsal tentacular cirri longest, reaching back to chaetigers VII–X.

Proboscis with pair of semi-transparent amber jaws, each with ca. ten teeth (up to ca. 15 teeth in dissected jaw, Fig. 2G). Dark pigmentation present on surface of proboscis (in particular, ventral surface) of four males of Malaysian specimens (Fig. 4B, C). Conical paragnaths present on maxillary ring (Fig. 4B, C); number of paragnaths and their arrangement on each area of everted proboscis as follows (Table 1): area I: 3–10, scattered and unequal; area II: 8–19, in two arched rows, markedly large paragnaths with sharply tapering and curved tip present in anterior and middle positions; area III: 32–50, in three or four rows of transversely elongated bands; area IV: 6–16, in triangular patch with markedly large paragnaths present in middle and posterior positions. Oral ring with no or few minute paragnaths; number of paragnaths on each area are as follows (Table 1): area V: none; area VI: none; area VII–VIII: 0–2, in transverse row. Total number of paragnaths 58–124. Pair of small nipple-like round papillae usually visible in right and left of area VI, as those in atokes (Fig. 2B).

***Male*** bodies divided into three regions (Fig. 3A): anterior (pre-natatory), middle (natatory), and posterior (post-natatory) regions; parapodia of pre-natatory and post-natatory regions similar to those of atokes (Fig. 6A, D).

Male pre-natatory region with 18–25 chaetigers, with dorsal cirri of first seven or eight chaetigers thickened mainly at base, and with ventral cirri of first 5–7 chaetigers thickened throughout (Fig. 7A). Neuropodial heterogomph falcigers (Fig. 6G) present in few chaetigers of pre-natatory region, appearing from chaetigers XV–XXI, or completely absent.

Male natatory region constituting of 30–56 chaetigers, with parapodia markedly modified (Figs 6B, 7B); round lobes newly present on upper and lower base of ventral cirri, appearing from chaetigers XIX–XXVI to chaetigers LII–LXXIII. Neuropodial postchaetal lobe developing into large round flat lamella with or without small triangular protrusion on lateral edge in almost same range of chaetigers; dorsal cirri frequently serrated on lower edge, slightly elongated; ovoid lobe newly present on upper base of dorsal cirri; all parapodial ligules and lobes enlarged as thin lamellae (Figs 6B, 7B). Epitokous paddle-like natatory chaetae (Figs 6H, 7E) appearing from chaetigers XXII–XXVIII to chaetigers LIV–LXXVIII, substituting atokous chaetae (Fig. 6E–G) completely in most of middle natatory region, and incompletely in few anteriormost and posteriormost chaetigers of this region (with atokous chaetae remaining there); blade of epitokous paddle chaetae semi-transparent, flat and wide, with minutely serrated edge on one side, and tapering tip.

Male post-natatory region constituting 13–64 chaetigers, with unmodified parapodia (Figs 3A, 6D); neuropodial heterogomph falcigers absent. Pygidium with pygidial rosette.

***Females*** with unmodified parapodia throughout, except for with dorsal cirri of first 4–8 chaetigers thickened mainly at base, and with ventral cirri of first 4–8 chaetigers slightly thickened throughout (Figs 3B, 5A, 6C, 7C, D). Epitokous paddle chaetae present together with atokous chaetae in both notochaetae and neurochaetae in middle body from chaetigers XXVI–XXXV to chaetigers XLVI–LII. Neuropodial heterogomph falcigers usually appearing from chaetigers XV–XVIII to chaetigers LIV–LXXII. Pygidium without pygidial rosette. Few eggs (full-grown oocytes) remained in coelom of females; eggs spherical, 100–140 µm in diameter in fixed specimens.

In both sexes, body wall of epitokes thin. Small slits on body wall of ventral surface at base of parapodia present in middle and posterior chaetigers of females (Fig. 5D).

##### Variation.

Paragnath numbers in epitokes from Thailand and Malaysia and atokes from Thailand are summarised together with the atokes from the previous studies in Table 1.

In three specimens, an epitokous female (PMBC 20732) and two atokes (PMBC 21211, 21212), a few heterogomph falcigers were present in the lower neurochaetae of chaetiger 1, whereas falcigers were usually absent in the anterior chaetigers (at least first 10 chaetigers) in the present study, as reported in the previous studies on *N. glandicincta* (Southern 1921; Lee and Glasby 2015; Ibrahim et al. 2019).

The papilla-like base of paragnaths in area III was not conspicuous in the ethanol-fixed epitokous materials.

**Table 1.** Variation in number of paragnaths of epitokes of *Neanthes glandicincta* collected from two estuaries in the coast of Peninsular Malaysia in the present study, in comparison with data of atokes in the present and previous studies.

Locality (no. of specimens examined)	Body width (mm)	Body length (mm)^1^	No. of total chaetigers^1^	Number of paragnaths^2^							Total^4^	References
				I	II^3^	III	IV^3^	V	VI^3^	VII–VIII		
Epitokes												
Songkhla Lagoon, Thailand (6)	1.2–2.1	17–34	68–117	8.0±2.8	14.2± 4.3	41.0± 7.9	11.2±3.5	0	0	0	98.4±27.9	Present study
				(3–10)	(8–19)	(32–50)	(6–16)	(0–0)	(0–0)	(0–0)	(58–124)	
Kuala Ibai, Malaysia (10)	1.6–3.2	33–43	62–123	5.3±1.8	14.8±3.0	41.5±6.2	11.2±1.6	0	0	0.3±0.7	95.8±11.3	Present study
				(3–8)	(8–19)	(32–50)	(9–13)	(0–0)	(0–0)	(0–2)	(74–113)	
Atokes												
Songkhla Lagoon, Thailand (10)	1.2–2.5	27–85	86–122	7.0±2.4	15.2±1.6	48.3±5.0	12.1±1.3	0	0.1±0.3	0.1±0.3	107.3±9.2	Present study
				(4–11)	(12–17)	(38–55)	(10–14)	(0–0)	(0–1)	(0–1)	(94–119)	
Eastern coast of Peninsular Malaysia (23)^5^	0.7–2.0	15–70	114–132	8.8±3.0	16.7±1.8	50.1±5.8	13.5±1.8	0	0.2±0.4	0.04±0.2	117.7±11.4	Ibrahim et al. (2019)
				(3–13)	(13–20)	(39–58)	(11–17)	(0–0)	(0–1)	(0–1)	(94–137)	
Nine sites in Singapore (54)^6^				9.0±3.4	17.3±2.5	49.2±7.2	14.1±2.5	0	0.1±0.3	1.2±2.1	120.1±13.9	Lee and Glasby (2015)
				(0–17)	(11–23)	(35–63)	(10–22)	(0–0)	(0–1)	(0–8)	(93–148)	
Maungmagan in Myanmar (8)^6,7^				5.8±3.9	13.1±2.0	41.3±9.7	14.0±2.9	0	0	0	101.3±19.9	Lee and Glasby (2015)
				(2–14)	(11–17)	(30–60)	(11–20)	(0–0)	(0–0)	(0–0)	(80–138)	
Calcutta in India (1)				10	12	38	7	0	1	2	90	Lee and Glasby (2015)
Near Calcutta in India (26)^8^		88	123	10	(10–13)	50	(10–12)	0	(0–1)	Up to 7		Southern (1921)

^1^ Data from complete specimens. ^2^ Mean±SD (range). ^3^ Larger value at a left or right side. ^4^ All total with numbers from both sides of areas II, IV and VI. ^5^ Pooled data from 3 sites, including two atokes collected from Kuala Ibai where epitokes were obtained in the present study. ^6^ Calculated based on the individual data shown in table 3 in Lee & Glasby (2015). ^7^ A part of syntypes of *Ceratonereis burmensis* Monro, 1937. ^8^ Original description of Nereis (Nereis) glandicincta Southern, 1921.

##### Habitat.

Intertidal and shallow subtidal bottoms of sandy or muddy sediment in the estuaries, where the salinity of ambient water widely ranges from 18 to 32 psu at Kuala Ibai in Malaysia (see below), and from 1 to 33 psu in the coast of the lower reaches of Songkhla Lagoon (Angsupanich and Rakkheaw 1997; S Angsupanich, unpublished data).

##### Geographic distribution.

India, Myanmar, Singapore, the east coast of Malay Peninsula (Malaysia and Thailand). Based on Southern (1921), Fauvel (1932, 1939, 1953), Misra (1999), Lee and Glasby (2015), Ibrahim et al. (2019), and the present study.

##### Remarks.

The morphological characteristics of present Thailand and Malaysian specimens of swimming epitokes agreed well with the atokes collected from Thailand in the present study and also the atokes previously described from India (Southern 1921; Lee and Glasby 2015), Myanmar (Monro 1937; Lee and Glasby 2015), Singapore (Lee and Glasby 2015), and Malaysia (Ibrahim et al. 2019), except for their epitokous modification of parapodia and chaetae in the middle body, and enlarged eyes. However, we found that a few falcigers were exceptionally present in the lower neurochaetae of chaetiger 1 in three Thailand specimens, unlike the previous descriptions of this species and also the diagnosis of *Neanthes glandicincta* species complex (Ibrahim et al. 2019). Therefore, the diagnosis of this species and the *Neanthes glandicincta* species complex should be amended here from Ibrahim et al. (2019) to allow for the occasional presence of falcigers in chaetiger 1.

The Indian specimens described as *N. glandicincta* by Misra (1999) seem to belong correctly to this species if Misra’s description “Notosetae homogomph spinigers and homogomph falcigers” is a mistake. The Chinese specimens described as *N. glandicincta* by Wu et al. (1985) seem to belong to an undescribed species of another member of the *Neanthes glandicincta* species complex because they differ from all other members of this species group in the absence of notopodial prechaetal lobe. Both atokous and epitokous specimens collected from southern China and identified as *Ceratonereis burmensis* by Wu et al. (1985) do not seem to belong to *N. glandicincta*; atokes with a lesser number of paragnaths seem to belong to *N. wilsonchani*, according to Lee and Glasby (2015) and the key of Ibrahim et al. (2019), whereas an epitoke with an indented anterior margin of the prostomium seemed to belong to *Ceratonereis*.

### Reproductive period and swimming behaviour of epitokes in Kuala Ibai in Malaysia

**Figure 8.**
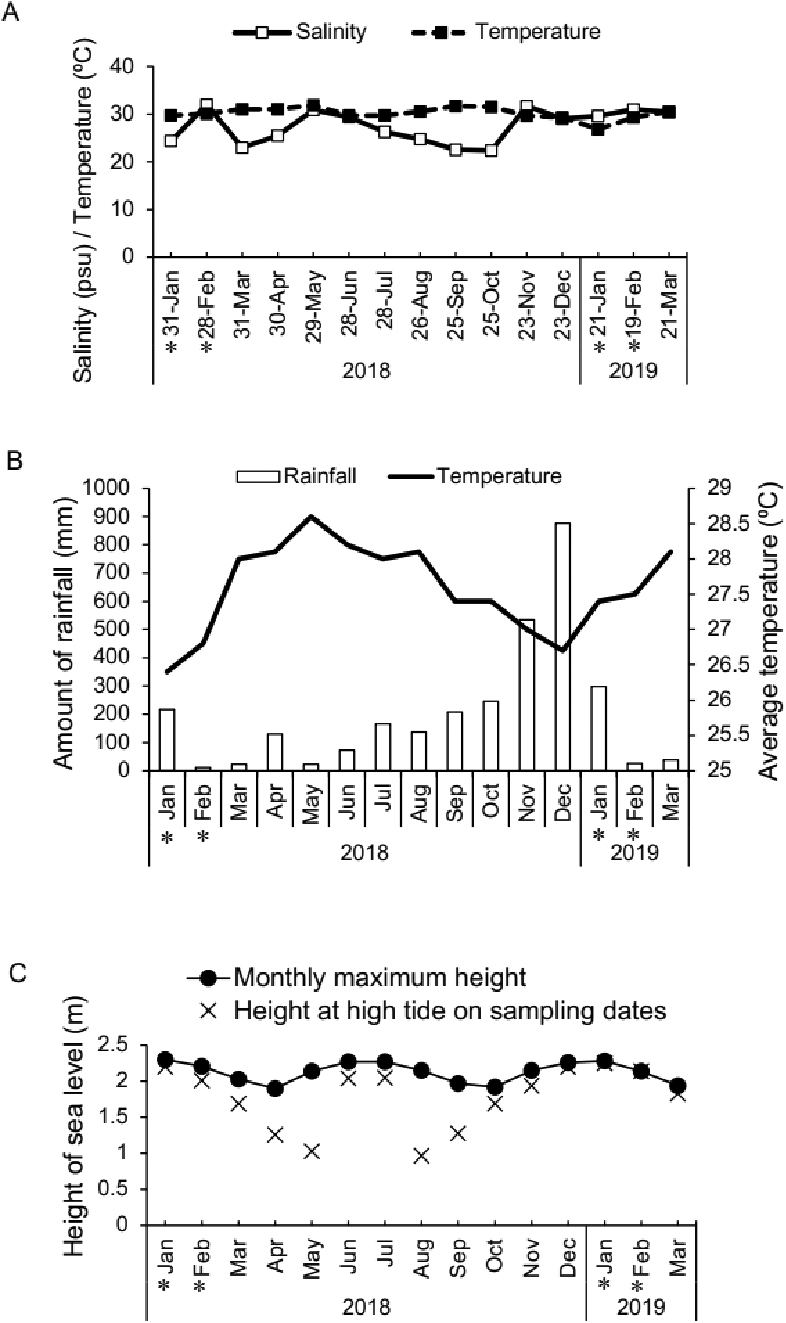
Seasonal changes of environmental parameters in Kuala Ibai, Malaysia during the sampling period **A** monthly changes in the salinity and temperature of the surface water at the sampling site (represented by our data at the end of each month) **B** monthly changes in the amount of rainfall and average air temperature at Kuala Ibai based on the data of Malaysian Meteorological Department **C** monthly changes of the maximum height of sea level at high tide (closed circles), with the height of sea level at high tide on each sampling date (x marks), based on the data of Worldwide Tides and Currents Predictor (2018). The asterisks indicate the months when the swimming epitokes of *Neanthes glandicincta* appeared.

A total of ten swimming epitokes of *Neanthes glandicincta* was collected during high tide at night (mostly within one hour before or after high tide, 20:45–23:49) around new moon or full moon in January and February during our 15-month sampling period from January 2018 to March 2019 in Kuala Ibai, Malaysia (Table 2). During this period, the water temperature (and salinity) varied in the range of 28–32 °C (18–32 psu) at the sampling site (Fig. 8A); salinity (24–32 psu) was relatively high, and the temperature was relatively low (28–30 °C) in January and February. The lowest and highest monthly amount of rainfall was recorded in February (11 mm) and December (877 mm) in 2018, respectively, with the general tendency that the average air temperature is relatively high in the dry season from March to August (28.0–28.6 °C), and relatively low in the rainy season from October to January (26.7–27.4 °C) at Kuala Ibai, according to the weather data of the Malaysian Meteorological Department (2019) (Fig. 8B).

**Table 2.** Occurrence of reproductive swimming of *Neanthes glandicincta* in Kuala Ibai, Terengganu.

**Date**	**Age of moon**	**Time of night high tide (Height of sea level)^1^**	**Sunset**	**Duration of observation**	**Catch time of epitokes (no. of inds. and sex^2^)**
2018					
31-Jan [2]^3^	14.0 (○)^4^	21:29 (2.20 m)	19:17	19:30–22:00	20:45 (1M),
					21:35 (1M)
28-Feb [3]	12.3 (○)	20:23 (2.01 m)	19:20	19:20–22:00	20:50 (1M)
					21:05 (1M)
					21:15 (1F)
02-Mar	14.3 (○)	21:36 (1.99 m)	19:20	19:30–22:00	19:20
31-Mar	13.6 (○)	20:53 (1.69 m)	19:16	19:23–22:30	19:16
30-Apr	14.0 (○)	20:27 (1.26 m)	19:13	19:10–22:30	19:13
15-May	29.0 (●)	19:30 (1.14 m)	19:14	19:31–02:00	19:14
29-May	13.6 (○)	19:09 (1.03 m)	19:16	19:10–22:30	19:16
28-Jun	14.3 (○)	09:40 (2.04 m)	19:23	19:25–04:00	19:23
13-Jul	0.0 (●)	09:27 (2.19 m)	19:25	19:25–23:00	19:25
28-Jul	15.0 (○)	09:24 (2.05 m)	19:24	19:25–22:30	19:24
11-Aug	29.0 (●)	18:55 (0.89 m)	19:21	18:55–01:30	19:21
26-Aug	14.7 (○)	19:40 (0.97 m)	19:16	19:30–22:30	19:16
25-Sep	15.4 (○)	20:55 (1.28 m)	19:02	19:10–22:00	19:02
09-Oct	29.4 (●)	21:01 (1.38 m)	18:55	18:56–22:00	18:55
25-Oct	16.0 (○)	21:41 (1.69 m)	18:50	19:00–23:00	18:50
08-Nov	30.0 (●)	21:44 (1.80 m)	18:48	18:58–22:30	18:48
23-Nov	15.4 (○)	21:37 (1.94 m)	18:49	19:15–23:00	18:49
23-Dec	15.8 (○)	22:05 (2.20 m)	19:00	19:10–23:00	19:00
2019					
06-Jan [3]	29.9 (●)	22:02 (2.13 m)	19:07	19:20–23:00	21:30 (3M) around 10 s interval
07-Jan	1.0 (●)	22:35 (2.15 m)	19:08	19:30–23:00	
21-Jan [2]	15.0 (○)	21:49 (2.25 m)	19:14	19:18–00:00	22:39 (1M), 23:49 (1M)
28-Jan	22.0 (**Ͻ**)	01:10 (1.64 m)	19:16	19:18–23:30	
05-Feb	0.3 (●)	22:14 (2.04 m)	19:18	19:20–23:00	
19-Feb [1]^5^	14.3 (○)	21:22 (2.14 m)	19:20	19:35–23:35	22:10
21-Mar	14.5 (○)	21:18 (1.83 m)	19:18	19:20–01:00	

^1^ Based on data of Worldwide Tides and Currents Predictor (2018). ^2^ M: male, F: female. ^3^ Number of epitokes found. ^4^ ○: within three days before or after full moon; ●: within three days before or after new moon; **Ͻ**: last quarter. ^5^ Specimen is not available, due to failure of catching it.

On the other hand, based on the daily tidal records in Kuala Terengganu (Worldwide Tides and Currents Predictor 2018), the monthly maximum height of the sea level at high tide was highest in June–July and December–January (2.26–2.28 m), and lowest in April and October (1.90–1.92 m) (Fig. 8C).

Male epitokes swam fast with a circular motion around artificial light. On 28 February 2018, two swimming males first appeared around half an hour after the high tide, followed by the occurrence of a swimming female ca. one hour after the high tide (Table 2). On 6 January 2019, three swimming males were collected at approximately 21:30 with an interval of ca. 10 seconds for each collection, but not followed by any swimming female. On 19 February 2019, an actively swimming adult was found just after the high tide at night but could not be collected.

### Mating behaviour and early development under laboratory culture in Thailand

In the cement ponds where the Thailand atokous specimens had been reared for several months, a pair of male and female epitokes were found swimming simultaneously out of the sediments and spawned in the water at 0:15 am on 28 February, and two more pairs of epitokes spawned at 1:55 am on 2 March 2006. In all three pairings, the male swam for a longer duration (ca. 30 min) than the female (ca. five min) and started swimming earlier than the female. At spawning, the male swam around the female. After spawning, the spent worms of both sexes sank to the bottom.

**Figure 9.**
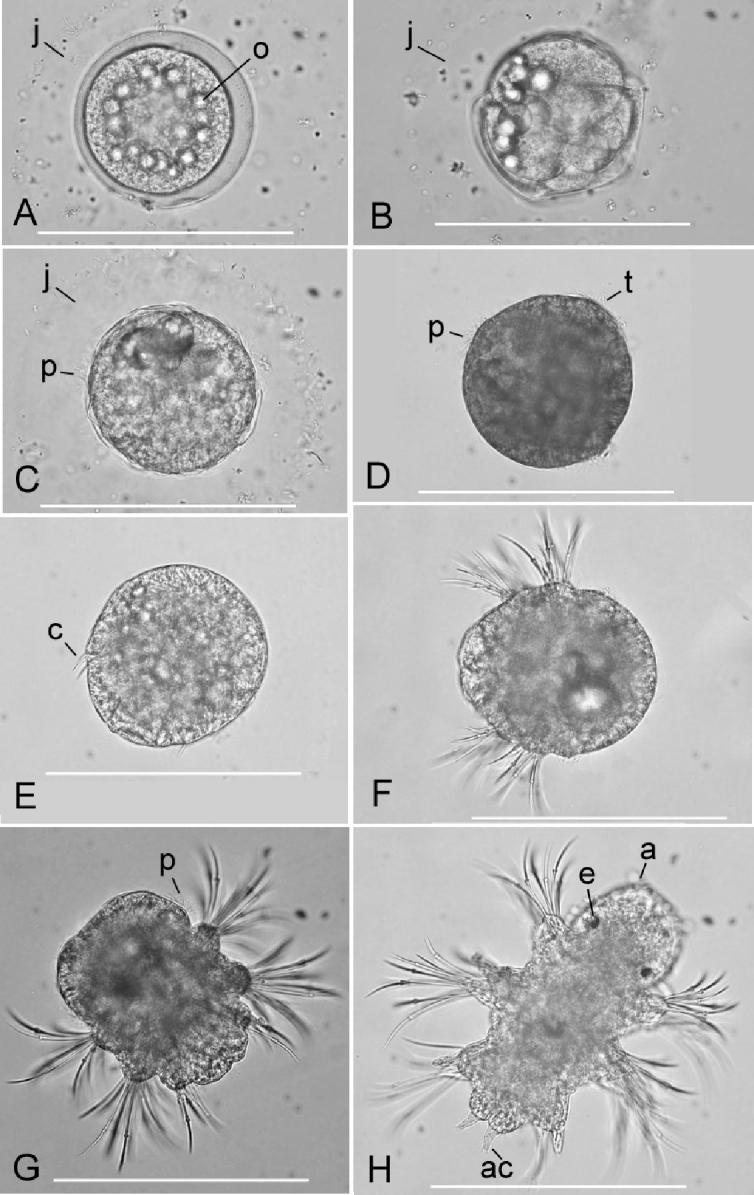
Early development of *Neanthes glandicincta* (Southern, 1921) after fertilisation in the laboratory. The material from the Lower Songkhla Lagoon, Thailand **A** fertilised egg surrounded by a jelly layer (j), 10 min after fertilisation; many sperm were trapped in the jelly layer; lipid (oil) drops (o) surrounded the germinal vesicle **B** 4-cell stage, 1 h and 10 min after fertilisation **C** early trochophore stage, 7 h and 30 min after fertilisation; ciliary movement of the prototroch (p) began within the jelly layer **D** free-swimming trochophore larva just after hatching out of the jelly layer, 8 h after fertilisation; ciliary bands of the prototroch and telotroch (t) were present **E** free-swimming early-metatrochophore larva, 20 h after fertilisation; two pairs of chaetal tufts (c) were present **F** free-swimming 2-chaetiger late-metatrochophore larva, 21 h after fertilisation; two pairs of chaetal tufts well developed **G** free-swimming early 3-chaetiger nectochaeta larva, 22 h after fertilisation; three pairs of chaetal tufts were developed; the prototroch and lipid drops remained in the anterior body **H** demersal late 3-chaetiger nectochaeta larva, 48 h after fertilisation; a pair of eyes (e), antennae (a), and anal cirri (ac) appeared. Lipid drops disappeared. Scale bars: 0.2 mm.

The eggs (fully-grown oocytes) just after fertilisation were relatively transparent and contained ca. 20 lipid drops surrounding the nucleus (germinal vesicle) (Fig. 9A). Successfully fertilised eggs formed a transparent cortex of 8–15 µm thickness beneath the egg surface, and a jelly layer ca. 50 µm thickness outside the egg surface (Fig. 9A). The embryonic development through 2-cell and 4-cell stages (Fig. 9B) up to the trochophore stage (Fig. 9C) progressed within the jelly layer. Trochophores hatched out of the jelly layer 8–9 h after the fertilisation, entering a free-swimming larval life (Fig. 9D). Approximately one day (20–23 h) after fertilisation, the larvae became early metatrochophores (Fig. 9E) and 2-chaetiger late metatrochophores (Fig. 9F), which were slightly elongated, posteriorly with two pairs of chaetal tufts. The larvae became early 3-chaetiger nectochaetes 22–27 h after fertilisation and began to enter a demersal life around the bottom layer, with a slow swimming behaviour (Fig. 9G). Approximately two days (48 h) after fertilisation, the larvae became late 3-chaetiger nectochaetes in which a pair of eyes and antennae in the prostomium, and a pair of anal cirri in the pygidium appeared (Fig. 9H); all of them crawled on the bottom surface, entering a benthic life as juveniles. Several large lipid drops, which probably originated from the smaller ones of the unfertilised eggs, were contained within the anterior body during the early larval stages but disappeared before the late 3-chaetiger nectochaeta stage.

## Discussion

### Epitokous metamorphosis and swimming behaviour of epitokes

In the present study, the reproductive characteristics of *Neanthes glandicincta* were examined using spawning fully mature adults in the field and laboratory culture. The results revealed that the typical epitokous metamorphosis to heteronereid form occurred in only males, including the enlargement of eyes, marked modification of parapodia, and complete substitution of atokous chaetae by natatory paddle-like chaetae in the natatory region. Whereas, only a partial epitokous metamorphosis occurred in mature females, including the enlargement of eyes, and incomplete substitution of atokous chaetae by natatory paddle-like chaetae with no modification of parapodia in the middle body.

Our findings well agreed with the previous reports of Fauvel (1939) who described that the male body divided into three parts with the epitokous modification occurring in the middle part (beginning at chaetiger XX) based on the specimens collected from Singapore. The present findings also agree with Monro (1937), who described that the eyes were markedly enlarged, the body was divided into three parts, and the epitokous modification of parapodia occurred in the middle part (beginning at chaetiger XXI), with the anterior and posterior parts remain unmodified, based on several males (a part of type material of *Ceratonereis burmensis*) collected from off Bombay, India. Lee and Glasby (2015) also described almost the same morphology of epitokous males in which the unmodified anterior part consisted of 20–22 chaetigers, and thereafter parapodia were modified bearing crenulate dorsal cirri in the mid-body.

The epitokous metamorphosis of certainly full-mature females is first described in the present study. In contrast to our result, Lee and Glasby (2015) described that ‘epitokous females’ had modified parapodia; same as those of males except for the absence of crenulate dorsal cirri based on the two specimens that were probably not fully matured. At present, the reason for the different findings is unknown.

The swimming behaviour of the epitokes of *N. glandicincta* was also first described in the present study, based on the field and laboratory observations. We found that both mature males and females spawn in estuarine waters while swimming at night high tide around the new moon and full moon in January and February in the field and that males swam longer than females before the paired mating behaviour and spawning occurred in laboratory conditions.

The sex ratio was heavily biased towards males (nine males: one female) in the field. The larger male proportion seems to be caused by males commencing swimming earlier and for a longer duration than females and thus can be collected more easily, as suggested in the reproductive swarming of the estuarine nereidids, *Hediste japonica* and *H. diadroma* (Hanafiah et al. 2006).

The typical epitokous metamorphosis to a heteronereid form in males seems to be significant, serving to increase the swimming ability of male epitokes, which need to swim at high speed to escape from predators and for a longer time to meet a female for successful spawning. On the other hand, the inconspicuous epitoky without parapodial modification in females seems to correspond to the short swimming duration of female epitokes.

As for spawning, Chan (2009) reported that the gametes are shed from the body wall in *N. glandicincta*. This is supported by our finding that ruptures existed in the body wall of the ventral surface of parapodia of spent worms after spawning.

The result of our monthly night sampling for swimming epitokes in Kuala Ibai indicates that the Malaysian population of *N. glandicincta* has a reproductive period from January to February, with an annual life cycle. This period roughly agrees with the period from February to March when we could collect mature swimming epitokes under the laboratory culture of the Thailand population in the present study. A similar reproductive period of this species has been suggested by Lee and Glasby (2015) who collected epitokes from mud flats in Singapore in December (four individuals), January (one), and April (one) of which some of their materials were not fully matured.

The east coast of the Malay Peninsula faces the South China Sea, where the northeast monsoon is dominant in a period from October to early March, whereas the southwest monsoon is dominant from late May to September (Akhir et al. 2011). The rainfall amount is usually the highest during the early northeast monsoon season (November and December) (Fig. 8B; Malaysian Meteorological Department 2018). The reproductive period of *N. glandicincta* coincides with the timing just after the rainy season. On the other hand, the reproductive period of this species also coincides with a period (one of two annual peaks), with the highest sea level at high tide, when the seawater can enter the uppermost reaches of estuaries (Fig. 8C). Ascertaining the environmental factors that affects the determination of the reproductive period in tropical estuaries would be an interesting future study.

Fig. 8C indicates that our choice of the sampling dates in April, May, August, and September in 2018 was inappropriate because the sea level at night on the high tides during these sampling dates was considerably less than the monthly maximum height of the sea level. Therefore, we cannot rule out the possibility that the swimming epitokes may appear in these months.

Fauvel (1932) found the 'subepitokous' males of *N. glandicincta*, which were collected from Vizagapatam on the eastern coast of India (near the type locality of this species) in May to June 1926. This suggests that the reproductive season in the type locality of this species may be different from that in the eastern Malay Peninsula. Whether the different reproductive seasons between geographically separated local populations could cause reproductive isolation between them, leading to speciation, is another interesting topic of study.

Fauvel (1932) also found no epitokous modification in a few females full of eggs and a fragment of a male filled with sperm among the many specimens collected from India and Thailand (Taléh-Sap = Songkhla Lagoon). Therefore, this suggests that some cryptic species may coexist with *N. glandicincta*.

### Early development

The early development of *Neanthes glandicincta* is first described in the present study (Fig. 9). The result demonstrated that the relatively small eggs (100–140 μm in diameter) formed a jelly layer just after fertilisation, and developed into trochophores, which hatched out of the jelly layer, entering a free-swimming larval life. This process of the embryonic development is almost the same as the relatively small (130–170 μm in diameter) eggs of the temperate estuarine nereidid, *Hediste diadroma* Sato & Nakashima, 2003 (Kagawa 1955, as *Neanthes japonica*; Sato and Tsuchiya 1991: small-egg type of *N. japonica*; Sato 1999: the small-egg form of *N. japonica*; Tosuji and Sato 2006; Sato 2017) in which a jelly layer formed just after fertilisation by the secretion of jelly substance from numerous cortical alveoli arranged in the egg surface layer (cortex) (Sato and Osanai 1986: *N. japonica*). Another temperate estuarine species, *H. japonica* (Izuka, 1908) with a relatively large egg diameter (180–210 μm) has a similar development also (Izuka 1908; Tosuji and Sato 2006; Sato 2017). However, the development of *N. glandicincta* could successfully progress under a relatively low salinity (15 psu); in contrast to that of *H. diadroma* and *H. japonica*, which both had a favourable salinity range of 22–30 psu and could not develop under 15 psu salinity (Sato and Tsuchiya 1987: small-egg type of *N. japonica*; Tosuji and Sato 2006). The tolerance of developing embryos against low salinity in *N. glandicincta* seems to be adaptive for its reproduction within an estuary with variable salinity.

The result indicates that the free-swimming larval phase in *N. glandicincta* is relatively short (two days in 25–30 °C): the shift from planktic to demersal larvae and the larval settlement on the bottom as crawling juveniles occur during the 3-chaetiger nectochaeta stage, when the lipid drops (maternal nutrients) disappear. On the other hand, in *H. diadroma* which has a catadromous life history (Sato 2017; Kan et al. 2020), the larval settlement occurred at the 5- to 8-chaetiger nectochaeta stage after a relatively long larval phase (more than 30 days in 15 °C), though the planktic larval life shifted to the demersal one, together with the disappearance of lipid drops during the 3-chaetiger nectochaeta, as in *N. glandicincta* (Tosuji and Sato 2006; Kan et al. 2020). The short period of planktic larval phase in *N. glandicincta* may be adaptive for preventing larvae from being washed out to sea.

These results indicate that the life cycle of *N. glandicincta* may be usually completed within an estuary with a limited larval dispersal ability.

## Supplementary Material

XML Treatment for Neanthes glandicincta
